# Fewer bat passes are detected during small, commercial drone flights

**DOI:** 10.1038/s41598-021-90905-0

**Published:** 2021-06-01

**Authors:** Gabrielle Ednie, David M. Bird, Kyle H. Elliott

**Affiliations:** Department of Natural Resource Science, McGill University, Ste-Anne-de-Bellevue, QC H9X 2E3 Canada

**Keywords:** Animal behaviour, Behavioural ecology, Conservation biology

## Abstract

Advances in technological capabilities, operational simplicity and cost efficiency have promoted the rapid integration of unmanned aerial vehicles (UAVs) into ecological research, providing access to study taxa that are otherwise difficult to survey, such as bats. Many bat species are currently at risk, but accurately surveying populations is challenging for species that do not roost in large aggregations. Acoustic recorders attached to UAVs provide an opportunity to survey bats in challenging habitats. However, UAVs may alter bat behaviour, leading to avoidance of the UAV, reduced detection rates and inaccurate surveys. We evaluated the number of bat passes detected with and without the presence of a small, commercial UAV in open habitats. Only 22% of bat passes were recorded in the presence of the UAV (0.23 ± 0.09 passes/min) compared to control periods without the UAV (1.03 ± 0.17 passes/min), but the effect was smaller on the big brown bat/silver-haired bat (*Eptesicus fuscus*/*Lasionycteris noctivagans*) acoustic complex. Noise interference from the UAV also reduced on-board bat detection rates. We conclude that acoustic records attached to UAVs may inaccurately survey bat populations due to low and variable detection rates by such recorders.

## Introduction

Unmanned aerial vehicles (UAVs), also known as drones, are rising in popularity and the technology is quickly becoming more cost-effective and energy-efficient^1–3^. As a result, UAVs are now quickly being integrated into scientific studies, including environmental research^2^. In many cases, the technology is used because UAVs allow surveys to be conducted remotely, eliminating the safety risk associated with traditional aerial surveys and reducing the required energy, cost, and time input from the researcher^3–5^. UAVs also facilitate access to previously inaccessible areas, providing information on species that occupy hard-to-access habitats^4,6^.

Although UAVs are increasingly used to survey animal populations, avoidance and aggressive behaviours by animals may reduce their effectiveness^2,4,7^, resulting in inaccurate population counts^4,7^. Furthermore, aggressive behaviour poses a risk of damage to the drone and/or injury to the study species^4^. Nonetheless, behavioural responses to UAVs are highly species-specific^2,7–12^. For example, raptorial bird species exhibit varying levels of aggression toward UAVs hovering over their nests^8^ and seabirds respond to UAVs only at sites where they regularly encounter aerial predators^7^. Manatees respond more readily to UAVs than dolphins, and dolphins respond more readily when alone than in small groups, implying that the noise and security associated with group-living may diminish responses to UAVs^9^. Indeed, even species that show no behavioural responses to drones may nonetheless mount a stress response, as documented for bears^13^. As such, understanding how UAVs affect species and their behaviour is critical before undertaking surveys.

As nocturnal species, bats (order: Chiroptera) are difficult to survey visually, and thus, the most common way to identify echolocating species is by listening to their echolocation calls used to locate prey during flight^14–16^. Identifying bats by the echolocation calls they emit is often an effective and minimally invasive method to study these species^14–16^. Such data are typically collected through acoustic inventories^14–16^ that detect bat passes, defined as a sequence of echolocation pulses, which are analyzed to identify species detected^15–17^. Many species spend substantial time hunting above the tree canopy where current echolocation recording devices cannot efficiently reach^6,18^. The research opportunities brought on by the emergence of UAVs is heralding the rapid development of UAVs as survey tools for these flying mammals^6,19–21^. Previous work demonstrating the effectiveness of UAVs for detecting bats provided qualitative support for an absence of an effect of UAVs on bat surveys^6,20^. While these studies demonstrate that bats can be detected by recorders on UAVs, they occurred at sites, such as cave entrances, where bats were forced to fly past the UAV^6,20,21^. It is unclear if detections are biased by avoidance behaviour under conditions where bats are able to avoid the UAV, such as surveys away from roosts attempting to associate bat density with foraging habitats.

Our study aims to assess the behavioural response and detectability of bats to UAVs (rotary-wing quadcopters) equipped with a small, commercial acoustic bat detector. Our goal was to test a proposed simple UAV-based surveying tool (similar to^6,20^) that citizen scientists or wildlife biologists could use to survey bats in remote areas without requiring an advanced pilot’s license. We conducted two separate experiments. First, we hypothesized that bats avoid UAVs, and thus, we predicted that we would detect more bat passes without UAVs than with UAVs present. Second, we considered how bat detection rates are influenced by UAV interference by assessing the detectability of echolocation in the presence of a UAV. We placed a bat detector at varying distances on the ground from the machine and examined qualitatively the distance at which the signal was no longer distinguishable from background noise.

## Materials and methods

### Site information

The study was conducted at the Kenauk Institute, an environmental research site, in western Quebec in July 2018, 2019 and 2020. All surveys occurred between 21h30 and 00h00 at night, with location and time of day randomized for each date of testing. Testing did not occur during inclement weather (rain or winds above 10 km/h). In 2018, during an initial field season, we surveyed bat populations using a traditional method (transect-based surveys) to determine which species were present. Six transects lasting 1.5 h each were laid out, and surveyed three times per season; three transects were located in open-canopy areas, and three were located in rugged, closed-canopy areas. Every 200 m, a flag marked a sampling point where we completed a 2-min static inventory using an Anabat SD2 (Titley Scientific, Columbia, MO). In this pilot study used to develop the main study, we observed all eight species known in Quebec, including the eastern red bat (*Lasiurus borealis*; 0.005 passes detected per minute in open-canopy habitat; 0.001 in closed-canopy), hoary bat (*Lasiurus cinereus*; 0.002 passes detected per minute in open-canopy habitat; 0.006 in closed-canopy) and tri-coloured bats (*Perimyotis subflavus*; 0.026 passes detected per minute in open-canopy habitat; none in closed-canopy). Species in the *Eptesicus fuscus*/*Lasionycteris noctivagans* acoustic complex were the most abundant (0.075 passes detected per minute in open-canopy habitat; 0.018 in closed-canopy) followed by *Myotis* species (*Myotis leibii, Myotis septentrionalis, Myotis lucifugus;* 0.075 passes detected per minute in open-canopy habitat; none in closed-canopy). Due to small sample sizes per species and because manual identification using spectrographic analyses can be unreliable for the differentiation of some bat species^22^, we pooled several bat species that had similar spectrograms into complexes. We pooled the big brown bat (*Eptesicus fuscus*) and the silver-haired bat (*Lasionycteris noctivagans*), and the *Myotis* species: little brown bat (*Myotis lucifugus*), northern long-eared Myotis (*M. septentrionalis*), and eastern small-footed bat (*M. leibii*)^22^. Therefore, these species are grouped together in analyses to minimize identification errors^22^. The big brown bat and silver-haired bat form the EPNO complex whereas the *Myotis* species form the MYSP complex. We identified to species the hoary bat (LACI), red bat (LABO), and tri-coloured bat (PESU)^22^. We identified bat passes visually using the output from the Anabat in the Anabat Insight software^17,23^.

### Detection efficiency

**Figure 1:**
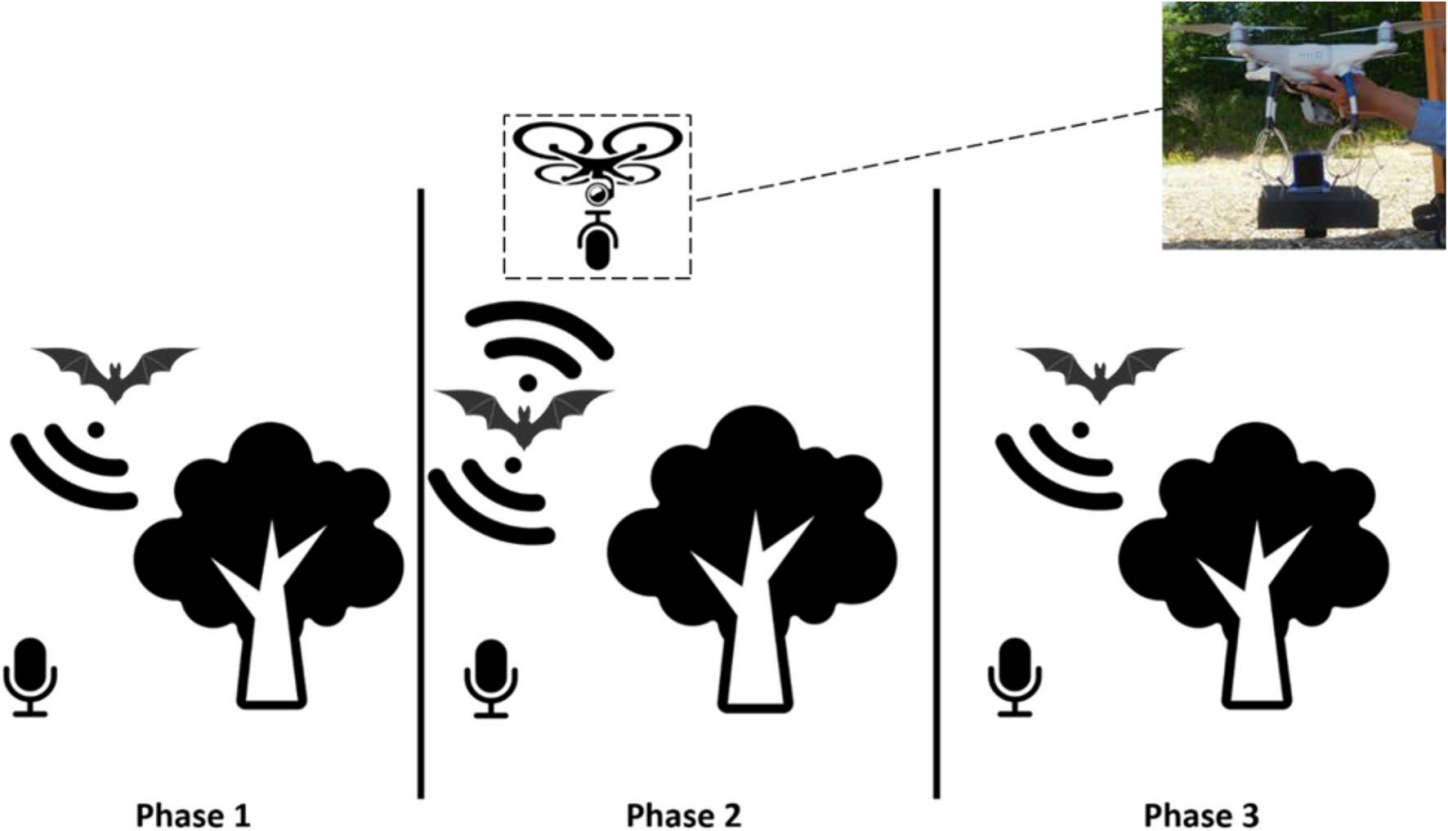
Illustration of the three phases of the experiment design. A photograph of the UAV setup used in Phase 2 is presented in the top right corner. The setup consists of an Echometer Touch bat detector from Wildlife Acoustics and 2-inch Sonoflat acoustic foam from Auralex attached to a DJI Phantom 4 quadcopter using zip ties. (Images by Julian Herzog, Symbolon, FontAwesome retrieved from https://commons.wikimedia.org. Picture taken by the author).

Because total bat passes per minute were seven times higher in open-canopy habitats than in closed-canopy habitats, in 2019 we focused our surveying efforts in relatively open habitats. The Anabat (420 g) is too large to attach to a drone, thus in 2019 and 2020, we used Echometer Touch bat detectors (20 g; Wildlife Acoustics, Maynard, MA), commercially available and inexpensive detectors, attached to iPod 7 s (88 g; Apple Inc., Cupertino, CA). We do not directly compare between surveys done with the Anabat and the Echometer Touch, but merely used the 2018 Anabat surveys as a guide for expected bat species and distributions in 2019 and 2020. The UAV used was a commercially available Phantom 4 quadcopter from DJI (1.3 kg, DJI Technology Co. Inc., Shenzhen, China). To reduce sound interference from the drone, which could reduce the detection range of the instrument, we placed a 2-in. Sonoflat acoustic foam (Auralex, Indianapolis, IN) divider between the recorder and the drone, as recommended by past studies^19,21^ (Fig. 1).

In both 2019 and 2020, we surveyed in three phases: (1) a 5-min recording from the ground without UAV; (2) a 5-min recording while the detector was attached to the UAV using zip ties and carabiners and while the UAV was manually flown in a 10–15 m diameter circle at canopy height (5––10 m above the pilot), depending on the survey site; and (3), identically to Phase 1, a 5-min recording taken from the ground without UAV (Fig. 1). The ground recorder, used sparsely in 2019 and consistently in 2020, was 1 m above the ground during phase 2. Based on surveys in 2018, seven sites were identified as having higher relative activity and were repeatedly monitored in 2019 and 2020 for bat activity. Of the seven study sites, five were located next to bodies of water and four were located near buildings; all were located in open areas. Open spaces and bodies of water are preferred hunting grounds for most bat species^18^, and make for an easier and safer drone flight. An additional bat detector (Echometer Touch 2, Wildlife Acoustics, Maynard USA) was used on the ground during Phase 2 to simultaneously monitor bat passes from the air and from the ground, to indicate whether bats were present but not detected due to UAV noise interference. In 2020, ten surveys were conducted with Echometer Touch 2 recorders on (1) the UAV, (2) on the ground, and (3) at a control site > 1 km from the current site. Control sites were only used in 2020. Because different bat detectors, as well as different classification software, detect and identify bats at different rates, we do not directly compare among different detectors or software^24,25^. In 2019, we used the Kaleidoscope software to identify bats automatically. We removed false identifications manually. In 2020, we used the Kaleidoscope software to identify all bats automatically. We also identified all passes visually and blind to the classification from Kaleidoscope. By classifying all bats using both software and visual identification, we aimed to determine whether our results were robust to identification technique.

Data were collected beyond Phase 1 if the site had a bat density above three passes per 5 min (2019: N = 24 without ground detector; N = 5 with ground detector; 2020: N = 10 with ground detector; all sample sizes refer to experiments that included Phases 2 and 3). If insufficient bat activity was recorded at a given site after a 5-min period, data collection moved on to the next site, and data from that site was excluded from any analyses. Phase 1 was done to ensure there was an established bat presence, and to maximize sampling. The length of each phase was extended to 10 min if two passes were detected by the 5-min mark of Phase 1, allowing for the collection of more data, while maintaining the time proportions of each phase. While this process, necessary logistically to obtain a sufficient sample size, could lead to more bats detected during Phase 1, there should be no impact on Phase 3 compared to Phase 2, and thus, we used Tukey tests to examine Phase 3 relative to Phase 2, as well as Phase 1 compared with both other phases^26^.

Each drone flight was performed by two field technicians: a pilot and an assistant. The UAV pilot held a basic operations pilot certificate for a small remotely-piloted aircraft system, visual line-of-sight (certificate number PC1917023611) in accordance with federal regulations enforced by Transport Canada. The assistant held the bat detector during Phases 1 and 3. During Phase 2, the assistant acted as the drone’s elevated launching and landing pad as the additional equipment obstructing the UAV’s landing gear. For take-off, they held the UAV upright above their head and gradually let go as the UAV gained altitude. For landing, the pilot gradually decreased the altitude of the drone until the landing gear was safely grasped by the assistant, who then held the UAV above their head until the propellers stopped moving. All methods were carried out in accordance with the guidelines of the Canadian Council for Animal Care. All experimental protocols were approved by McGill University animal care committee under protocol 2015-7599 and complied with the ARRIVE guidelines for animals.

Statistical analyses were conducted using R 3.6.0 base package^26^. Generalized linear models (glm, Poisson distribution) were performed to determine the effect of phase (i.e., 1, 2, and 3) and detector location (detector on the UAV or on the ground) on the total number of bat passes. Tukey tests were then used to determine what phases and locations were significantly different from one another. To assess interspecific variation in detectability, the difference between the mean detection rate for Phase 1 and 3 and the detection rate in Phase 2 were calculated by species for each survey. A glm was then performed on the difference in detectability by species ([Average of Phases 1 and 3 − Average of Phase 2]–Species). Species were divided into four categories: MYSP (*Myotis* species complex), EPNO (big brown bat/silver-haired bat complex), LABO (eastern red bat), and LACI (hoary bat). No tri-coloured bats were detected, and are therefore absent from analyses. Detection phases were also divided into four categories in relation to the UAV flight: Phase 1 (pre-flight), Phase 2 from UAV-based detection (during flight), Phase 2 from ground-based detection (ground), and Phase 3 (post-flight).

### Detection capacity

To estimate the degree to which technological limitations affected the results gathered during the first experiment, a second experiment was conducted to estimate the impact of propeller-noise interference on the range of the bat detector. An Audio Generator SGA-8200 (Circuit-Test, Burnaby, Canada), connected to an Ultra Sound Advice S55/6 amplifier and loudspeaker (Ultra Sound Advice, London, UK) set to broadcast a 40 kHz sine wave at 40 dB SPL_A_ @ 1 m, the highest dB setting, was used to replicate the high amplitude ultrasound reached by most bat species during their echolocation calls^22^. The Echometer Touch bat detector was moved away from the speaker along a measuring tape until the ultrasonic frequency could no longer be detected by the microphone. The procedure was then repeated with the detector attached to the flying UAV. As ambient sound perception cannot be evaluated when the microphone is attached to the UAV, the spectrogram on the Echometer Touch cellphone app (Wildlife Acoustics) connected to the detector was recorded with the screen video recording feature of the iPod 7 (Apple). These recordings were taken as the drone and bat detector were flown slowly along the ground to three distances (10 m, 15 m, 20 m) away from the ultrasound generator to better approximate the detection range. The videos were later visually assessed qualitatively by estimating the distance at which the signal from the speaker could no longer be distinguished from the noise interference of the drone.

To quantify the spectral overlap of the drone with echolocation pulses, a spectral analysis of three 15 s recordings were performed using Avisoft SASLab Pro 4.40 (Avisoft Bioacoustics, Berlin Germany). These recordings included the drone flying, the drone motors running without propellers attached, and the ambient noise from the same location and time (control). Recordings were saved as 16 bit WAV files sampled at 256 kc/s and were normalized to 90% in SASLab Pro prior to parameterization. Spectrographs of those normalized recordings were generated using a Fast Fourier Transform length of 512 points, with a frame size of 100% and 75% overlap of Hann windows. This achieved a frequency resolution of 500 Hz and temporal resolution of 0.5 ms. Frequencies where noise was concentrated are evident from these spectrographs, but were confirmed by generating Logarithmic Power Spectra from each recording using Hann windowing achieving frequency resolution of 0.061 Hz. Noise is described at frequencies where the relative sound pressure level exceeded − 80 dB in those Power Spectra.

## Results

### Detection efficiency

**Figure 2:**
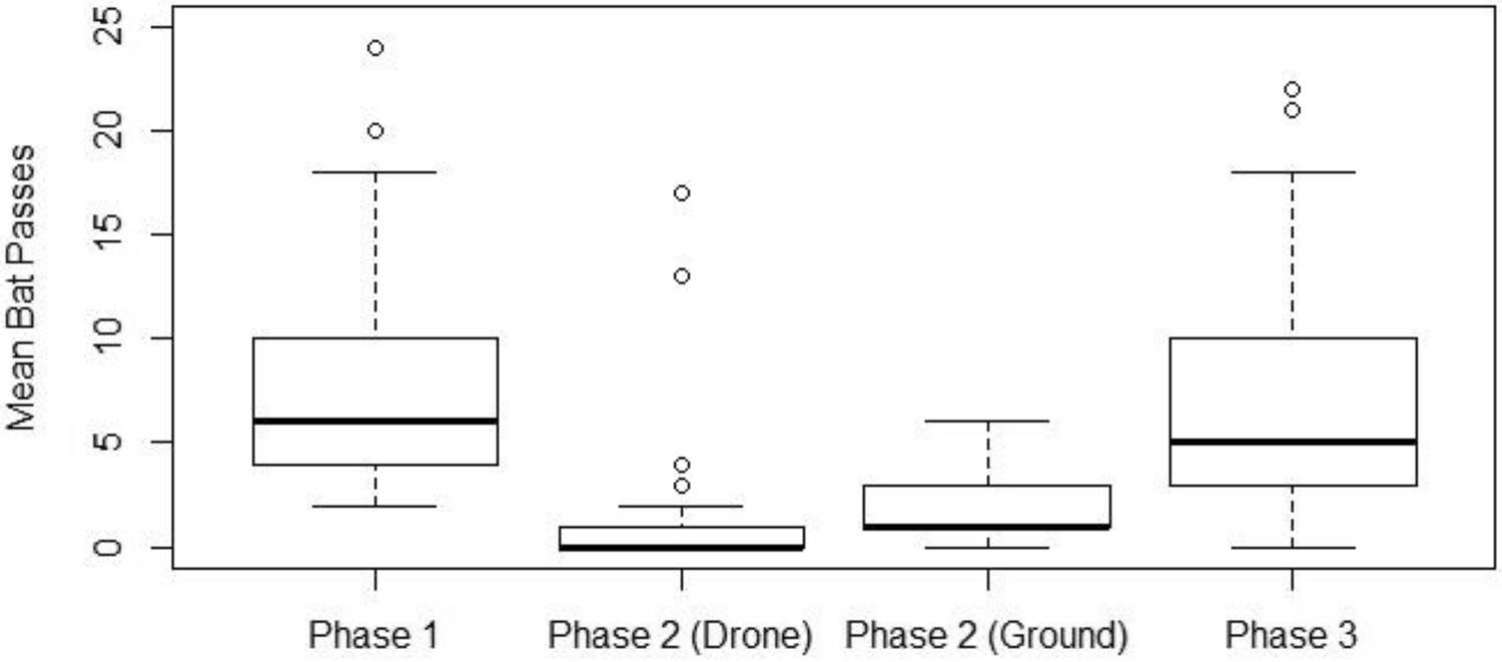
Mean bat passes per detection phase in 2019. The bold line in each box plot represents the median number of bat passes detected per detection phase. Phase 1 occurred prior to UAV flights, Phase 2 occurred during UAV flights either recorded on the ground or on the UAV (drone) and Phase 3 occurred after the UAV flights. The upper and lower extremities of each box represent the upper and lower quartiles, while the whiskers represent the smallest and largest non-outlier data points, which are represented by hollow points^26^.

The 2019 surveys (N = 29) revealed that phase (F_2,84_ = 14.2, p < 0.0001), but not detector location (F_1,7_ = 1.33, p = 0.29), was associated with the number of bat passes detected across all seven sites. There was no difference in bat pass detection rate between Phase 1 and Phase 3 (Tukey’s test, p = 0.86) or ground and during flight in Phase 2 (Tukey’s test, p = 0.62), but all other differences were statistically significant (Tukey’s test, p < 0.001; Fig. 2). Similarly, in 2020, phase was associated with the number of bat passes detected in the UAV (F_3,36_ = 6.39, P = 0.001), but not control (F_2,27_ = 0.82, P = 0.44) flights (N = 10). There was no difference in bat pass detection rate between Phase 1 and Phase 3 (Tukey’s test, p = 0.44) or ground and during flight in Phase 2 (Tukey’s test, p = 0.71), but all other differences were statistically significant (Tukey’s test, p < 0.01). Visual identification of bat passes did not change the significance of any of those results.

**Figure 3:**
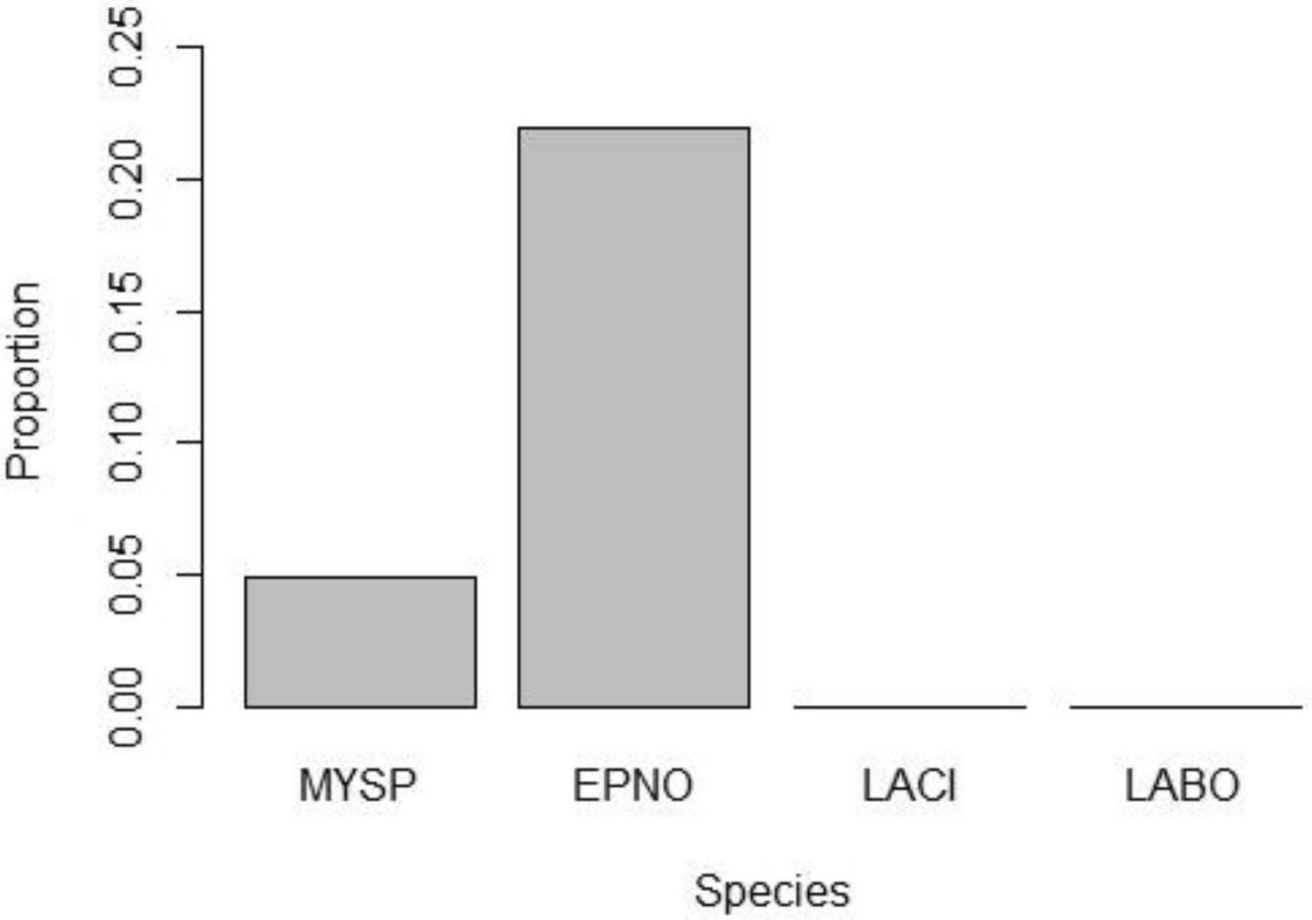
Proportion of bat passes detected per species group with and without a UAV. These rates were obtained by dividing the results of Phase 2 (drone flight) by the average of Phases 1 and 3 (before and after drone flights)^26^.

Species groups all yielded significant differences (χ^2^_3_, N = 29 = 57.21, p < 0.00001) between the mean detectability of Phases 1 and 3, and Phase 2. There were differences between LABO and EPNO (p < 0.001), LACI and EPNO (p < 0.001), MYSP and EPNO (p < 0.001), and MYSP and LABO (p = 0.006), but not LACI and LABO (p = 0.57) or MYSP and LACI (p = 0.19). The EPNO complex had the highest proportional number of bat passes during Phase 2 with 22% of the amount detected on average during Phase 1 and 3 (Fig. 3). Thus, the number of bat passes detected by UAV for all species (0.23 ± 0.09 passes/min) was below 23% of the number detected without the UAV (1.03 ± 0.17 passes/min; Fig. 2 converted in values per unit time).

### Detection capacity

**Figure 4:**
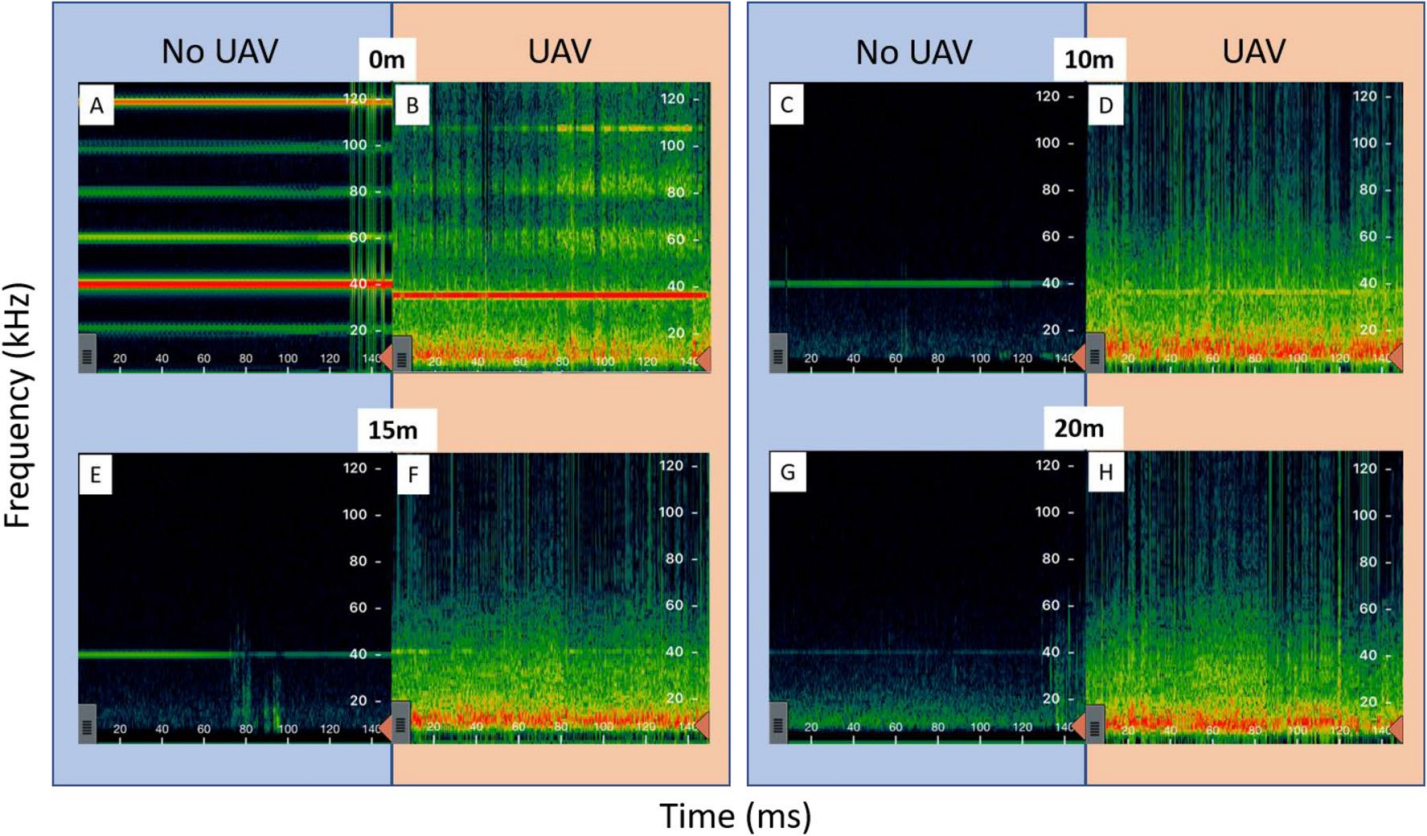
Visual comparison of signal detection range with ambient noise and UAV noise interference. Shading represents noise amplitude from low (green) to high (red). Panels inside the blue zones (**A**,**C**,**E**,**G**) represent samples taken under ambient noise interference, while panels inside the red zones (**B**,**D**,**F**,**H**) represent samples with UAV noise interference. Distance indicators represent the distance of the detector from the loudspeaker broadcasting ultrasounds. Bats present in our study area had calls typically between 25 and 80 Hz.

The range over which the Echometer Touch detected ultrasound varied when located on or off an active UAV. The signal emitted by the sine wave generator could be detected up to 28 m under ambient testing conditions with clear attenuation of the signal strength starting at 20 m. Comparatively, the range of detection decreased to approximately 15 m with UAV noise interference (Fig. 4).

**Figure 5:**
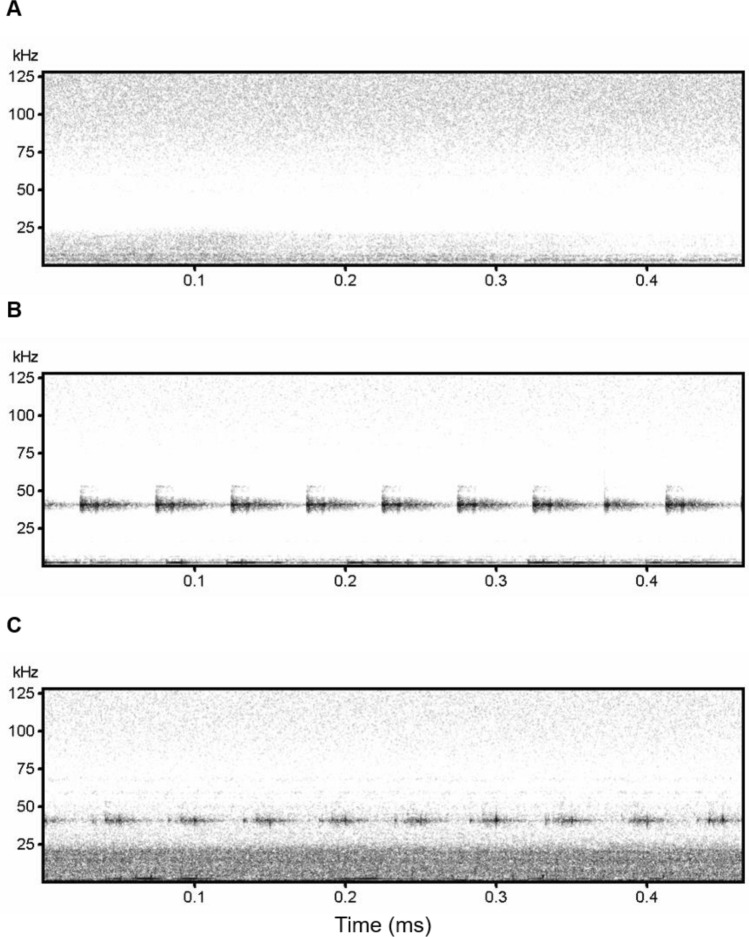
Spectrographs of possible noise interference sources from the unmanned aerial vehicle. Spectrographs of (**A**) ambient noise, (**B**) drone motor noise and (**C**) the flying drone. Spectrographs were generated in SASLab Pro.

Ambient noise at the time of recording was minimal, with no noise resolved between 25 and 60 kHz, and only light broadband noise above 60 kHz, and below 25 kHz, with concentrations around 4 kHz and 270 Hz (Fig. 5A). The drone motors alone produced relatively intense pulsatile noise centered around 40.5 kHz, and pronounced intermittent noise centered at 2.1 kHz (Fig. 5B). The flying drone produced rapidly pulsing noise concentrated around 40.5 kHz, and more consistent, loud broadband noise between 0 and 20 kHz (Fig. 5C).

## Discussion

In accordance with our hypothesis, fewer bat passes were detected during Phase 2 of the experiment, regardless of the position of the detector, with no difference during Phase 2 of the ‘control’ (no UAV present) recordings. Because the testing occurred over 15–30 min, with several tests each night, it is unlikely that the variation within each test, which were randomized, were due to systematic variation in time of day, weather or other factors. The reduction in bat detection rates during Phase 2 occurred for all species groups but was larger for the MYSP complex than the EPNO complex. This effect may be caused by the spectral overlap of the MYSP species echolocation calls with pronounced drone noise at 40.5 kHz. As the EPNO complex has lower average call frequencies with less spectral overlap, their detectability would not be compromised as severely by this noise. These findings contrast with another study that did not report any reduction in detection capabilities with their UAV model^6^. However, test sites in that previous study were located where large swarms of bats were obligated to pass right by the drone to exit a cave^6^. Moreover, our system, based on a 1.3 kg Phantom 4 UAV, was less than half the weight of the 3.3 kg Spreading Wings S900 used in the bat cave study (which also had a thermal imaging camera attached to the UAV), and, as such, produced at least 10 dB less noise^27^. Despite reduced size, noise and cost, our system would be ineffective in surveying bats due to the pronounced reduction in the number of bats recorded with UAV operation. Noise interference, often produced in the audible range (< 20 kHz), most severely impacts bat species emitting low-frequency calls (< 35 kHz)^28^. However, our results indicate that the EPNO complex was detected at a proportionally higher rate during Phase 2 of our sample period. This suggests that the EPNO complex was the least affected by the UAV, even though their average call frequency is 32 kHz^22^. Conversely, the species in the MYSP complex, which were present at proportionally lower rates during Phase 2, have an average call frequency of 40 kHz, well above the audible range^22^. These discrepancies were also observed by Luo et al.^29^.

Noise from the drone may also have impeded the ability of the bat detector to detect bats. Current commercially available detectors attached to the UAV used in our study appear incapable of picking up simulated bat echolocation calls much beyond 15 m (Fig. 4). With no noise interference, the same detector can record calls past 20 m. Also, many studies have found that detection rates are diminished at higher altitudes^19,21^ due in part to a reduction in bat densities at higher altitudes^19^ and by a reduction in the effective detection radius of microphones at high altitudes^21^. For microphones flying at 5 m, the effective detection radius is already reduced by half^21^. However, in our study, there was no significant difference in bat passes recorded by the drone and ground recorders during Phase 2. As a result, drone interference may reduce detection capacity, but behavioural avoidance is the most likely cause of the reduced bat detection rates in Phase 2. Indeed, it seems unlikely that a reduction in bat passes of 78–95% (i.e. 5–22% the number of passes detected, depending on species and including only common species) would occur due to a reduction in detection range from 20 to 15 m and drone noise interference concentrated at 40.5 kHz.

Fewer bat passes in the two most abundant bat groups occurred during Phase 2 of our experiment lead one to conclude that bats simply avoid places where UAVs are being flown. Past experiments that found bats tend to avoid loud noises because it may affect their foraging success would support this supposition^28,29^. However, one must bear in mind that our study had several limitations. Because the project aimed to produce a surveying tool that is reproducible by citizen scientists or wildlife biologists with limited UAV experience wishing to survey bats, we used a UAV and bat detector that were commercially available and affordable. A quieter UAV equipped with a bat detector capable of filtering out noise interference and/or with a longer range would likely improve detection rates. It might also be worthwhile applying the technique successfully used to census birds whereupon the detector was hung from the drone by a cord^30^. Also, our Phase 1 may have been inflated because we did not complete surveys with 0–2 bats detected in Phase 1 to limit time wasted surveying when no bats were present. However, given that Phase 1 and 3 were not different from one another, but strongly different from Phase 2, this issue could not have led to our results, as this bias in Phase 1 would have transferred to Phase 3 but not Phase 2.

In conclusion, while UAVs are being used to answer many novel questions in conservation science^7,24,25,30,31^, disturbance to wildlife from the drone is an important consideration for any conservation or related management application^2,4,7,8,10,13,32–34^. We concluded that bats in our study were less detectable around drones, at least in open habitats, likely due to the noise the machines generate. Until the technology involving both commercially available and affordable drones and bat detectors improves, UAVs may not be the most effective tools to perform aerial active or passive bat surveys due to their low and variable detectability.

## Supplementary Information

Supplementary Information.

## Data Availability

All data generated or analysed during this study will be included in this published article (and its “Supplementary information” files).
